# Prehospital STEMI Referral Systems and Sex-Related Bias in Canada: A National Survey

**DOI:** 10.1016/j.cjco.2022.05.006

**Published:** 2022-05-31

**Authors:** Laurie-Anne Boivin-Proulx, Christine Pacheco, Alexis Matteau, Samer Mansour, Brian J. Potter

**Affiliations:** a Centre Cardiovasculaire du Centre Hospitalier de l’Université de Montréal (CHUM), Montréal, Quebec, Canada; b Centre de Recherche du CHUM (CRCHUM), Montréal, Quebec, Canada; c Hôpital Pierre-Boucher, Université de Montréal, Montréal, Quebec, Canada

## Abstract

**Background:**

Prehospital electrocardiographic ST-elevation myocardial infarction (STEMI) diagnosis and prehospital cardiac catheterization laboratory activation have been shown to significantly reduce average treatment delay, and further standardization of such systems may help reduce sex-related treatment and outcome gaps. However, what types of prehospital STEMI activation systems are in place across Canada, and to what extent sex-based STEMI treatment disparities are tracked, is unknown.

**Methods:**

We conducted a national survey of catheterization laboratory directors between October 11 and December 25, 2021. Seventeen catheterization laboratory directors representing 6 community and 11 academic centres completed the survey (40% response rate).

**Results:**

: All responding centres use a prehospital STEMI diagnosis and cardiac catheterization laboratory activation system, and the majority (59%) rely on real-time physician oversight. Slightly less than half (47%) of percutaneous coronary intervention centres reported prospectively tracking sex-related differences in STEMI care, and only one respondent believed that a significant systemic sex-related bias was present in their prehospital STEMI referral system. Patient factors (symptom description or time to presentation; 23.5%) and limitations of electrocardiogram diagnosis of STEMI in women (23.5%) were cited most frequently as contributing to sex-related bias in STEMI referral systems. In contrast, implicit bias in the referral algorithm, prehospital provider bias, and physician bias were not considered important contributing factors.

**Conclusions:**

Although all responding centres employ prehospital activation systems, less than half tracked sex-related differences, and most respondents believed that no sex-related bias existed in their prehospital STEMI system.

## Résumé

**Contexte:**

Il a été démontré que le diagnostic préhospitalier par électrocardiogramme de l’infarctus du myocarde avec élévation du segment ST (STEMI) et l’activation du laboratoire de cathétérisme cardiaque préhospitalier permettent de réduire significativement le délai moyen de traitement, et un usage plus standardisé de ces systèmes pourrait contribuer à la réduction des écarts liés au sexe dans les traitements et les résultats de santé. Toutefois, on ignore quels types de systèmes d’activation préhospitaliers pour la prise en charge du STEMI sont en place à travers le Canada, ni dans quelle mesure les disparités de traitement liées au sexe font l’objet d’un suivi.

**Méthodologie:**

Nous avons mené un sondage national auprès des directeurs de laboratoires de cathétérisme entre le 11 octobre et le 25 décembre 2021. Notre questionnaire a été rempli par les directeurs de 17 laboratoires de cathétérisme, dont six centres communautaires et 11 centres universitaires (taux de réponse de 40 %).

**Résultats:**

Pour tous les centres répondants, un système de diagnostic préhospitalier du STEMI et d’activation de laboratoire de cathétérisme cardiaque était en place, dont la surveillance était assurée majoritairement par un médecin en temps réel (59 %). Un peu moins de la moitié des centres d’intervention coronarienne percutanée (47 %) ont rapporté faire un suivi prospectif des différences liées au sexe dans la prise en charge du STEMI, et un seul répondant a dit croire qu’un biais systématique lié au sexe était présent dans le système d’orientation préhospitalier pour les STEMI. Les facteurs liés aux patients (la description des symptômes ou le temps écoulé depuis leur apparition; 23,5 %) et les limites inhérentes au diagnostic du STEMI par électrocardiogramme chez les femmes (23,5 %) étaient les facteurs les plus fréquemment mentionnés comme sources de biais liés au sexe dans les systèmes d’orientation pour les STEMI. Par ailleurs, les biais implicites dans l’algorithme d’orientation et les biais des prestataires de soins préhospitaliers et des médecins n’étaient pas des facteurs contributifs jugés importants.

**Conclusions:**

Bien que des systèmes d’activation préhospitaliers soient en place dans tous les centres répondants, moins de la moitié d’entre eux ont rapporté faire le suivi des différences liées au sexe, et la plupart des répondants étaient d’avis que leur système préhospitalier de prise en charge des STEMI ne comportait pas de biais liés au sexe.

Women diagnosed with ST-elevation myocardial infarction (STEMI) continue to experience suboptimal treatment delays (TDs) and higher rates of adverse outcomes, compared to those for men.^1^ Prehospital electrocardiographic STEMI diagnosis and prehospital cardiac catheterization laboratory (CCL) activation have been shown to significantly reduce average TDs,^2^ and further standardization of STEMI diagnosis and CCL activation may help reduce sex-related treatment and outcome gaps.^3^^,^^4^ A recent retrospective analysis suggested that provider bias may be an important factor to consider, given that an automated system without real-time physician oversight was associated with a reduced sex-related gap in suboptimal first medical contact-to-device time (> 90 minutes).^3^ However, what types of prehospital STEMI activation systems are in place across Canada, and to what extent sex-based STEMI treatment disparities are tracked, is unknown. We therefore conducted a national survey of catheterization laboratory directors (N = 43) between October 11 and December 25, 2021.

## Methods

Seventeen catheterization laboratory directors, representing 6 community centres and 11 academic centres, completed the survey (40% response rate). Respondents were from Alberta (n = 3), British Columbia (n = 1), Ontario (n = 8), Quebec (n = 4), and Saskatchewan (n = 1)**.** All responding centres use a prehospital STEMI diagnosis and cardiac catheterization laboratory activation system, and the majority (59%) rely on real-time physician oversight. A similar proportion (65%) stated that real-time physician oversight is necessary to ensure the accuracy and appropriateness of prehospital CCL activation. Nearly all centres that did not use real-time physician oversight (n = 6 of 7; 86%) relied on automated electrocardiogram (ECG) interpretation. Eleven centres (65%) stated that they had formally evaluated the impact of establishing a prehospital system and deemed it to be associated with meaningful improvements in TD. Five (29%) incorporated a pharmacoinvasive strategy option (upfront fibrinolysis coupled with timely percutaneous coronary intervention [PCI] according to reperfusion criteria) as part of their prehospital STEMI referral system.

## Results

A high proportion of respondents agreed that the rate of ECG-inappropriate activations (86%) and false negatives (94%) should be ≤ 5%. However, the consensus level was low concerning an acceptable false-positive rate (ie, any activation resulting from an accurately identified elevation in the ST segment without a significant lesion in a corresponding artery or alteration in thrombolysis in myocardial infarction [TIMI] flow), with 6 (35.3%), 5 (29.4%), and 6 (35.3%) respondents believing that it should be ≤ 5%, 5%-10%, and 10%-15%, respectively.

**Figure 1:**
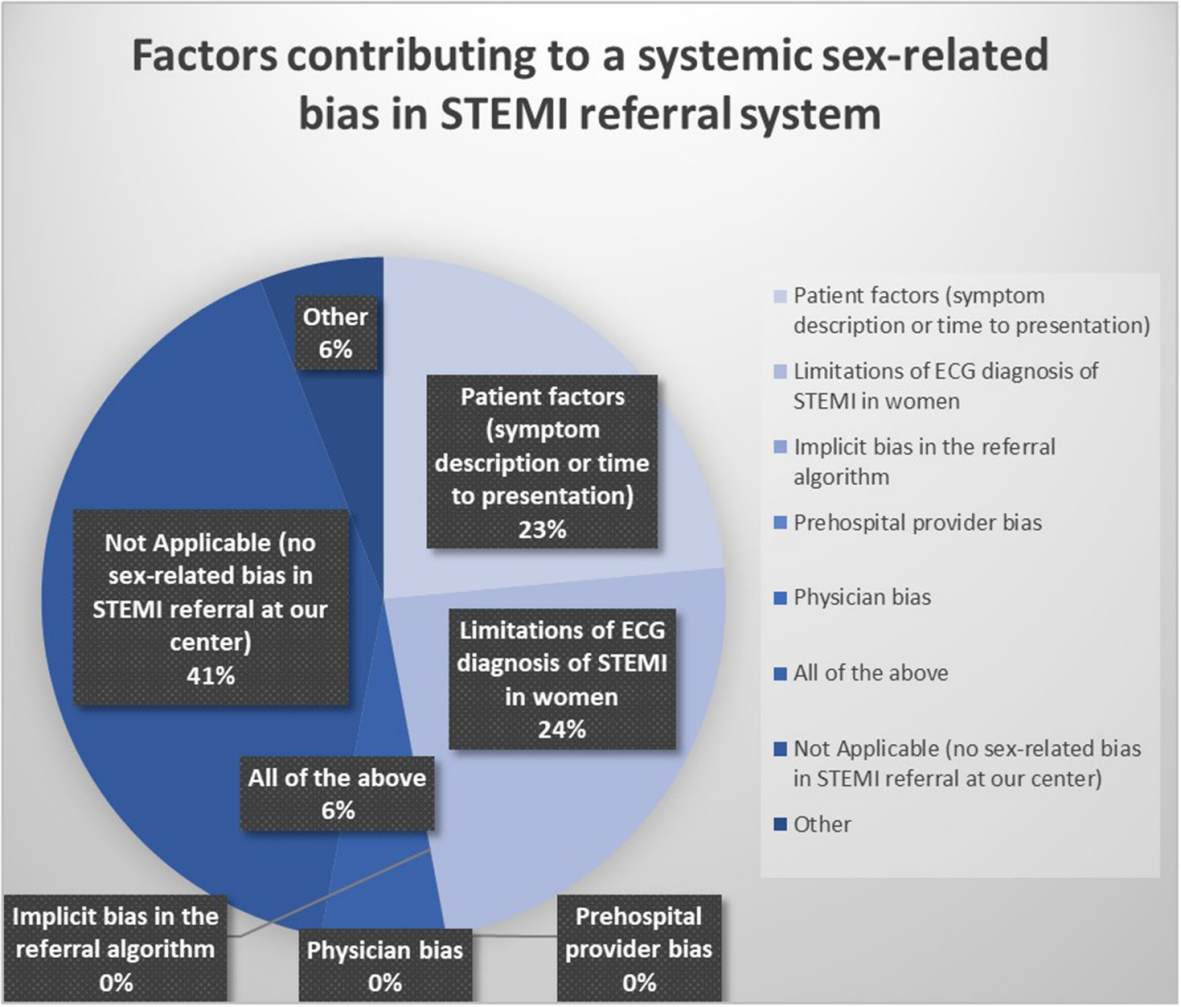
Factors contributing to a systemic sex-related bias in ST-elevation myocardial infarction (STEMI) referral system. ECG, electrocardiogram.

Slightly less than half of PCI centres (47%) reported prospectively tracking sex-related differences in STEMI care, and only one respondent believed that a significant systemic sex-related bias was present in their prehospital STEMI referral system. Patient factors (symptom description or time to presentation; 23.5%) and limitations of ECG diagnosis of STEMI in women (23.5%) were the items most frequently indicated as contributing to a sex-related bias in STEMI referral systems (Fig. 1). In contrast, implicit bias in the referral algorithm, prehospital provider bias, and physician bias were not considered important factors contributing to a sex-related bias. Nearly 90% of respondents do not believe that real-time physician oversight contributes to sex-related healthcare disparities in STEMI. However, in keeping with the 2021 American College of Cardiology/American Heart Association Chest Pain Management Guidelines, which state that the reduction of sex differences in treatment and outcomes should be an important future goal of research and clinical care,^5^ all respondents also stated that they were willing to contribute data to a national STEMI care research project examining sex-related bias.

## Discussion

The results of this survey of Canadian catheterization laboratories reveal several important findings. First, broad adoption of prehospital STEMI referral systems had occurred among responding centres and was associated with improvement in TD overall, in alignment with results in the literature.^2^ Second, nearly two-thirds of surveyed prehospital systems are physician-activated, and a similar proportion believe that physician oversight is essential for ensuring diagnostic accuracy. However, automated STEMI referral has been shown to have good accuracy in terms of minimizing both false-positive and ECG-inappropriate activations,^6^ but it has yet to be evaluated in the context of a system that includes a pharmacoinvasive strategy (used by nearly 30% of respondents). Third, given the body of evidence showing that women continue to experience suboptimal TD and worse outcomes more frequently than do men,^1^ 2 points of interest are that less than half of PCI centres currently track sex-related differences in STEMI care and almost all respondents believed significant systemic sex-related bias did not exist in their prehospital STEMI referral system. Moreover, nearly all respondents did not believe that prehospital provider or physician bias plays a role in sex-related bias, whereas a complex interplay among all these factors likely explains the persistent treatment and outcome gaps for women. Checklist-based approaches are known to reduce sex-based TD and outcome gaps in the treatment of STEMI and should be incorporated into prehospital STEMI referral systems to minimize potential healthcare provider bias.^4^ Artificial intelligence and machine learning can also contribute to reducing implicit bias originating from healthcare providers.^7^ Although artificial intelligence-guided interpretation of ECGs was shown to reliably diagnose STEMI,^8^ its use has yet to be evaluated in prehospital STEMI referral systems, *and its impact on mitigating treatment gaps in this setting remains unknown*.

## Conclusions

Although increasing female patients' awareness of their risk of cardiovascular disease and the range of possible STEMI symptoms will help reduce treatment and outcome gaps, rigorous assessment of STEMI referral systems for possible systemic bias is recommended. Specifically, although some overlap is present, sex-based treatment gaps prior to first medical contact may be considered to be primarily patient-driven, whereas those subsequent to first medical contact have to be attributed to deficiencies or bias related to system factors. Thus, the broad willingness among respondents to contribute data to a national assessment of the impact of STEMI referral systems on sex-related differences in management and outcomes should be seen in a very positive light and should encourage the Canadian Society of Cardiology to track system performance according to sex and for specific minority groups.
